# A telescope GWAS analysis strategy, based on SNPs-genes-pathways ensamble and on multivariate algorithms, to characterize late onset Alzheimer’s disease

**DOI:** 10.1038/s41598-020-67699-8

**Published:** 2020-07-21

**Authors:** Margherita Squillario, Giulia Abate, Federico Tomasi, Veronica Tozzo, Annalisa Barla, Daniela Uberti, The Alzheimer’s Disease Neuroimaging Initiative, Michael W. Weiner, Paul Aisen, Ronald Petersen, Jack R. Clifford, William Jagust, John Q. Trojanowki, Arthur W. Toga, Laurel Beckett, Robert C. Green, Andrew J. Saykin, John Morris, Leslie M. Shaw, Zaven Khachaturian, Greg Sorensen, Maria Carrillo, Lew Kuller, Marc Raichle, Steven Paul, Peter Davies, Howard Fillit, Franz Hefti, Davie Holtzman, M. Marcel Mesulam, William Potter, Peter Snyder, Tom Montine, Ronald G. Thomas, Michael Donohue, Sarah Walter, Tamie Sather, Gus Jiminez, Archana B. Balasubramanian, Jennifer Mason, Iris Sim, Danielle Harvey, Matthew Bernstein, Nick Fox, Paul Thompson, Norbert Schuff, Charles DeCarli, Bret Borowski, Jeff Gunter, Matt Senjem, Prashanthi Vemuri, David Jones, Kejal Kantarci, Chad Ward, Robert A. Koeppe, Norm Foster, Eric M. Reiman, Kewei Chen, Chet Mathis, Susan Landau, Nigel J. Cairns, Erin Householder, Lisa Taylor-Reinwald, Virginia Lee, Magdalena Korecka, Michal Figurski, Karen Crawford, Scott Neu, Tatiana M. Foroud, Steven Potkin, Li Shen, Kelley Faber, Sungeun Kim, Lean Tha, Richard Frank, John Hsiao, Jeffrey Kaye, Joseph Quinn, Lisa Silbert, Betty Lind, Raina Carter, Sara Dolen, Beau Ances, Maria Carroll, Mary L. Creech, Erin Franklin, Mark A. Mintun, Stacy Schneider, Angela Oliver, Lon S. Schneider, Sonia Pawluczyk, Mauricio Beccera, Liberty Teodoro, Bryan M. Spann, James Brewer, Helen Vanderswag, Adam Fleisher, Daniel Marson, Randall Griffith, David Clark, David Geldmacher, John Brockington, Erik Roberson, Marissa Natelson Love, Judith L. Heidebrink, Joanne L. Lord, Sara S. Mason, Colleen S. Albers, David Knopman, Kris Johnson, Hillel Grossman, Effie Mitsis, Raj C. Shah, Leyla de Toledo-Morrell, Rachelle S. Doody, Javier Villanueva-Meyer, Munir Chowdhury, Susan Rountree, Mimi Dang, Ranjan Duara, Daniel Varon, Maria T. Greig, Peggy Roberts, Yaakov Stern, Lawrence S. Honig, Karen L. Bell, Marilyn Albert, Chiadi Onyike, Daniel D’Agostino, Stephanie Kielb, James E. Galvin, Brittany Cerbone, Christina A. Michel, Dana M. Pogorelec, Henry Rusinek, Mony J. de Leon, Lidia Glodzik, Susan De Santi, Kyle Womack, Dana Mathews, Mary Quiceno, P. Murali Doraiswamy, Jeffrey R. Petrella, Salvador Borges-Neto, Terence Z. Wong, Edward Coleman, Allan I. Levey, James J. Lah, Janet S. Cella, Jeffrey M. Burns, Russell H. Swerdlow, William M. Brooks, Steven E. Arnold, Jason H. Karlawish, David Wolk, Christopher M. Clark, Liana Apostolova, Kathleen Tingus, Ellen Woo, Daniel H. S. Silverman, Po H. Lu, George Bartzokis, Charles D. Smith, Greg Jicha, Peter Hardy, Partha Sinha, Elizabeth Oates, Gary Conrad, Neill R. Graff-Radford, Francine Parfitt, Tracy Kendall, Heather Johnson, Oscar L. Lopez, Mary Ann Oakley, Donna M. Simpson, Martin R. Farlow, Ann Marie Hake, Brandy R. Matthews, Jared R. Brosch, Scott Herring, Cynthia Hunt, Anton P. Porsteinsson, Bonnie S. Goldstein, Kim Martin, Kelly M. Makino, M. Saleem Ismail, Connie Brand, Ruth A. Mulnard, Gaby Thai, Catherine Mc-Adams-Ortiz, Christopher H. van Dyck, Richard E. Carson, Martha G. MacAvoy, Pradeep Varma, Howard Chertkow, Howard Bergman, Chris Hosein, Sandra Black, Bojana Stefanovic, Curtis Caldwell, Ging-Yuek Robin Hsiung, Howard Feldman, Benita Mudge, Michele Assaly, Elizabeth Finger, Stephen Pasternack, Irina Rachisky, Dick Trost, Andrew Kertesz, Charles Bernick, Donna Munic, Kristine Lipowski, Masandra Weintraub, Borna Bonakdarpour, Diana Kerwin, Chuang-Kuo Wu, Nancy Johnson, Carl Sadowsky, Teresa Villena, Raymond Scott Turner, Kathleen Johnson, Brigid Reynolds, Reisa A. Sperling, Keith A. Johnson, Gad Marshall, Jerome Yesavage, Joy L. Taylor, Barton Lane, Allyson Rosen, Jared Tinklenberg, Marwan N. Sabbagh, Christine M. Belden, Sandra A. Jacobson, Sherye A. Sirrel, Neil Kowall, Ronald Killiany, Andrew E. Budson, Alexander Norbash, Patricia Lynn Johnson, Thomas O. Obisesan, Saba Wolday, Joanne Allard, Alan Lerner, Paula Ogrocki, Curtis Tatsuoka, Parianne Fatica, Evan Fletcher, Pauline Maillard, John Olichney, Owen Carmichael, Smita Kittur, Michael Borrie, T.-Y. Lee, Rob Bartha, Sterling Johnson, Sanjay Asthana, Cynthia M. Carlsson, Adrian Preda, Dana Nguyen, Pierre Tariot, Anna Burke, Nadira Trncic, Adam Fleisher, Stephanie Reeder, Vernice Bates, Horacio Capote, Michelle Rainka, Douglas W. Scharre, Maria Kataki, Anahita Adeli, Earl A. Zimmerman, Dzintra Celmins, Alice D. Brown, Godfrey D. Pearlson, Karen Blank, Karen Anderson, Laura A. Flashman, Marc Seltzer, Mary L. Hynes, Robert B. Santulli, Kaycee M. Sink, Leslie Gordineer, Jeff D. Williamson, Pradeep Garg, Franklin Watkins, Brian R. Ott, Henry Querfurth, Geoffrey Tremont, Stephen Salloway, Paul Malloy, Stephen Correia, Howard J. Rosen, Bruce L. Miller, David Perry, Jacobo Mintzer, Kenneth Spicer, David Bachman, Elizabether Finger, Stephen Pasternak, Irina Rachinsky, John Rogers, Dick Drost, Nunzio Pomara, Raymundo Hernando, Antero Sarrael, Susan K. Schultz, Laura L. Boles Ponto, Hyungsub Shim, Karen Ekstam Smith, Norman Relkin, Gloria Chaing, Michael Lin, Lisa Ravdin, Amanda Smith, Balebail Ashok Raj, Kristin Fargher

**Affiliations:** 1 DIBRIS, University of Genoa, 16146 Genoa, Italy; 2 Department of Molecular and Translational Medicine, University of Brescia, 25123 Brescia, Italy; 3 UC San Francisco, San Francisco, CA 94107 USA; 4 UC San Diego, La Jolla, CA 92093 USA; 5 Mayo Clinic, Rochester, MN USA; 6 UC Berkeley, Berkeley, San Francisco USA; 7 University of Pennsylvania, Philadelphia, PA 19104 USA; 8 USC, Los Angeles, CA 90032 USA; 9 UC Davis, Sacramento, CA USA; 10 10Brigham and Women’s Hospital/Harvard Medical School, Boston, MA 02215 USA; 11 Indiana University, Bloomington, IN 47405 USA; 12 Washington University St. Louis, St. Louis, MO 63110 USA; 13 Prevent Alzheimer’s Disease 2020, Rockville, MD 20850 USA; 14 Siemens, Erlangen, Germany; 15 Alzheimer’s Association, Chicago, IL 60631 USA; 16 University of Pittsburg, Pittsburgh, PA 15213 USA; 17 Cornell University, Ithaca, NY 14853 USA; 18 Albert Einstein College of Medicine of Yeshiva University, Bronx, NY 10461 USA; 19 AD Drug Discovery Foundation, New York, NY 10019 USA; 20 Acumen Pharmaceuticals, Livermore, CA 94551 USA; 21 Northwestern University, Chicago, IL 60611 USA; 22 National Institute of Mental Health, Bethesda, MD 20892 USA; 23 Brown University, Providence, RI 02912 USA; 24 University of Washington, Seattle, WA 98195 USA; 25 University of London, London, UK; 26 UCLA, Torrance, CA 90509 USA; 27 University of Michigan, Ann Arbor, MI 48109-2800 USA; 28 University of Utah, Salt Lake City, UT 84112 USA; 29 Banner Alzheimer’s Institute, Phoenix, AZ 85006 USA; 30 UUC Irvine, Orange, CA 92868 USA; 31 Johns Hopkins University, Baltimore, MD 21205 USA; 32 Richard Frank Consulting, Consulting, USA; 33 National Institute on Aging, Baltimore, MD USA; 34 Oregon Health and Science University, Portland, OR 97239 USA; 35 University of Alabama, Birmingham, AL USA; 36 Mount Sinai School of Medicine, New York, NY USA; 37 Rush University Medical Center, Chicago, IL 60612 USA; 38 Baylor College of Medicine, Houston, TX USA; 39 Wien Center, Miami Beach, FL 33140 USA; 40 Columbia University Medical Center, New York, NY USA; 41 New York University, New York, NY USA; 42 University of Texas Southwestern Medical School, Galveston, TX 77555 USA; 43 Duke University Medical Center, Durham, NC USA; 44 Emory University, Atlanta, GA 30307 USA; 45 University of Kansas Medical Center, Kansas City, KS USA; 46 University of Kentucky, Lexington, KY USA; 47 Mayo Clinic, Jacksonville, FL USA; 48 University of Rochester Medical Center, Rochester, NY 14642 USA; 49 Yale University School of Medicine, New Haven, CT USA; 50 McGill Univ. Montreal-Jewish General Hospital, Montreal, PQ H3A 2A7 Canada; 51 Sunnybrook Health Sciences, Toronto, ON Canada; 52 U.B.C. Clinic for AD & Related Disorders, Vancouver, BC Canada; 53 Cognitive Neurology-St. Joseph’s, London, ON Canada; 54 Cleveland Clinic Lou Ruvo Center for Brain Health, Las Vegas, NV 89106 USA; 55 Premiere Research Inst (Palm Beach Neurology), West Palm Beach, FL USA; 56 Georgetown University Medical Center, Washington, DC 20007 USA; 57 Stanford University, Stanford, CA 94305 USA; 58 Boston University, Boston, MA USA; 59 Howard University, Washington, DC 20059 USA; 60 Case Western Reserve University, Cleveland, OH 44106 USA; 61 Neurological Care of CNY, Liverpool, NY 13088 USA; 62 St. Joseph’s Health Care, London, ON N6A 4H1 Canada; 63 Dent Neurologic Institute, Amherst, NY 14226 USA; 64 Ohio State University, Columbus, OH 43210 USA; 65 Albany Medical College, Albany, NY 12208 USA; 66 Hartford Hospital Olin Neuropsychiatry Research Center, Hartford, CT 06114 USA; 67 Dartmouth-Hitchcock Medical Center, Lebanon, NH USA; 68 Wake Forest University Health Sciences, Winston-Salem, NC USA; 69 Medical University South Carolina, Charleston, SC 29425 USA; 70 Nathan Kline Institute, Orangeburg, NY USA; 71 University of Iowa College of Medicine, Iowa City, IA 52242 USA; 72 University of South Florida: USF Health Byrd Alzheimer’s Institute, Tampa, FL 33613 USA

**Keywords:** Machine learning, Alzheimer's disease, Genetics research

## Abstract

Genome–wide association studies (GWAS) have revealed a plethora of putative susceptibility genes for Alzheimer’s disease (AD), with the sole exception of APOE gene unequivocally validated in independent study. Considering that the etiology of complex diseases like AD could depend on functional multiple genes interaction network, here we proposed an alternative GWAS analysis strategy based on (i) multivariate methods and on a (ii) telescope approach, in order to guarantee the identification of correlated variables, and reveal their connections at three biological connected levels. Specifically as multivariate methods, we employed two machine learning algorithms and a genetic association test and we considered SNPs, Genes and Pathways features in the analysis of two public GWAS dataset (ADNI-1 and ADNI-2). For each dataset and for each feature we addressed two binary classifications tasks: cases vs. controls and the low vs. high risk of developing AD considering the allelic status of APOEe4. This complex strategy allowed the identification of SNPs, genes and pathways lists statistically robust and meaningful from the biological viewpoint. Among the results, we confirm the involvement of TOMM40 gene in AD and we propose GRM7 as a novel gene significantly associated with AD.

## Introduction

Alzheimer’s disease (AD) is the predominant form of dementia (50–75%) in the elderly population. Two forms of AD are known: an early-onset (EOAD) that affects the 2–10% of the patients and is inherited in an autosomal dominant way, with three genes *APP*, *PS1* and *PS2* mainly involved; a late-onset form (LOAD) that affects the vast majority of the patients in the elderly over 65s, whose causes remain still unknown^1^. Although LOAD has been defined as a multifactorial disease and its inheritance pattern has not been clarify yet, it is coming out the idea that it could be likely caused by multiple low penetrance genetic variants^2^, with a genetic predisposition for the patients and their relatives estimated of nearly 60–80%^2^.

The first well known gene associated to LOAD was *APOE*^3^. It encodes three known isoforms proteins (*APOE2*, *APOE3* and *APOE4*), with *APOE4* known to increase risk in familial and sporadic EOAD. This risk is estimated to be threefold and 15-fold for heterozygous and homozygous carriers respectively, with a dose-dependent effect on onset age^2^.

Large-scale collaborative GWAS and the International Genomics of Alzheimer’s Project have significantly advanced the knowledge regarding the genetics of LOAD^1^. Anyways, none of the new identified loci reached the magnitude of *APOEe4*, as predisposing risk factor for AD, with the majority of the hereditable component of AD remaining unexplained^4^. Several different but not mutually exclusive explanations of such failure could coexist: AD could be caused by the concerted action of independent genetic factors, each having a small effect size that require to adopt multivariate methods and to increase sample size^5^; or it could be caused by the concerted actions of multiple genes (again characterized by low effect size) that act inter-dependently in still undefined pathways, that would need a pathway-based approach, as done for other complex diseases^6^. Alternatively, AD could be caused by vary rare but highly penetrant mutations that might be identified through DNA sequencing^7^.

In order to explore the first two possible scenarios, in this study we proposed an alternative GWAS analysis strategy based on (i) multivariate methods and on (ii) a SNPs-Genes-Pathways ensamble, in order to guarantee the identification of correlated variables, and reveal the possible connections existing among the identified relevant variables at different, but biologically connected levels.

**Figure 1:**
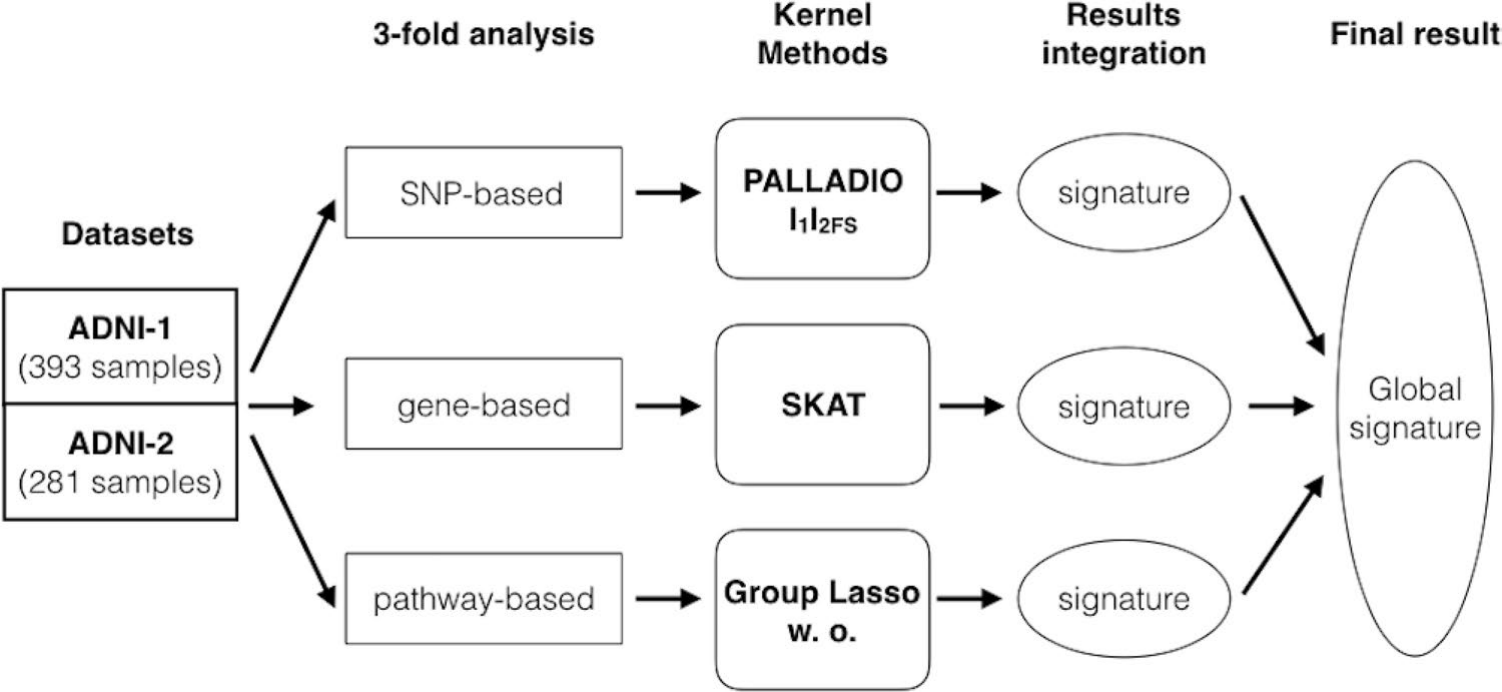
The alternative GWAS’s analysis strategy. ADNI-1 and ADNI-2 datasets were analyzed unimputed at the SNP, Gene and Pathway levels using three machine learning methods [i.e., l_1_l_2FS_ within PALLADIO framework, SKAT and Group Lasso with overlap (w. o.)]. The global signature represents the summary of the single integrated signatures identified within the proposed GWAS strategy.

Figure 1 depicts this alternative strategy. We analyzed both datasets at the SNPs, genes and pathways levels: in the SNPs analysis we used a multivariate methods named l1l2_FS_, in the genes analysis we used an association genetic test named SKAT and in the pathways analysis we considered Group Lasso with overlap. All these methods share the multivariate aspect, because they consider more features simultaneously (i.e., all the SNPs of one chromosome in the first analysis, all the SNPs belonging to one gene in the gene based analysis and all the pathways of one group in the pathway based analysis) differently from the univariate methods, such as the t-test, that evaluate the statistical association of each single feature at the time. The final purpose is to identify lists or signatures of possible causal SNPs, genes and pathways that considered together might provide a convincing picture of heritable factors in the LOAD pathogenesis.

In the Results section we show the signatures of SNPs, genes and pathways identified considering both the binary classification tasks, cases@controls and *APOEe4*, while in the discussion we comment the obtained results considering the possible integration of the signatures across the SNPs, genes and pathways levels and also across ADNI-1 and ADNI-2 dataset that we analyzed separately and considering only the genotype SNPs.

## Results

### SNP-based results

**Figure 2:**
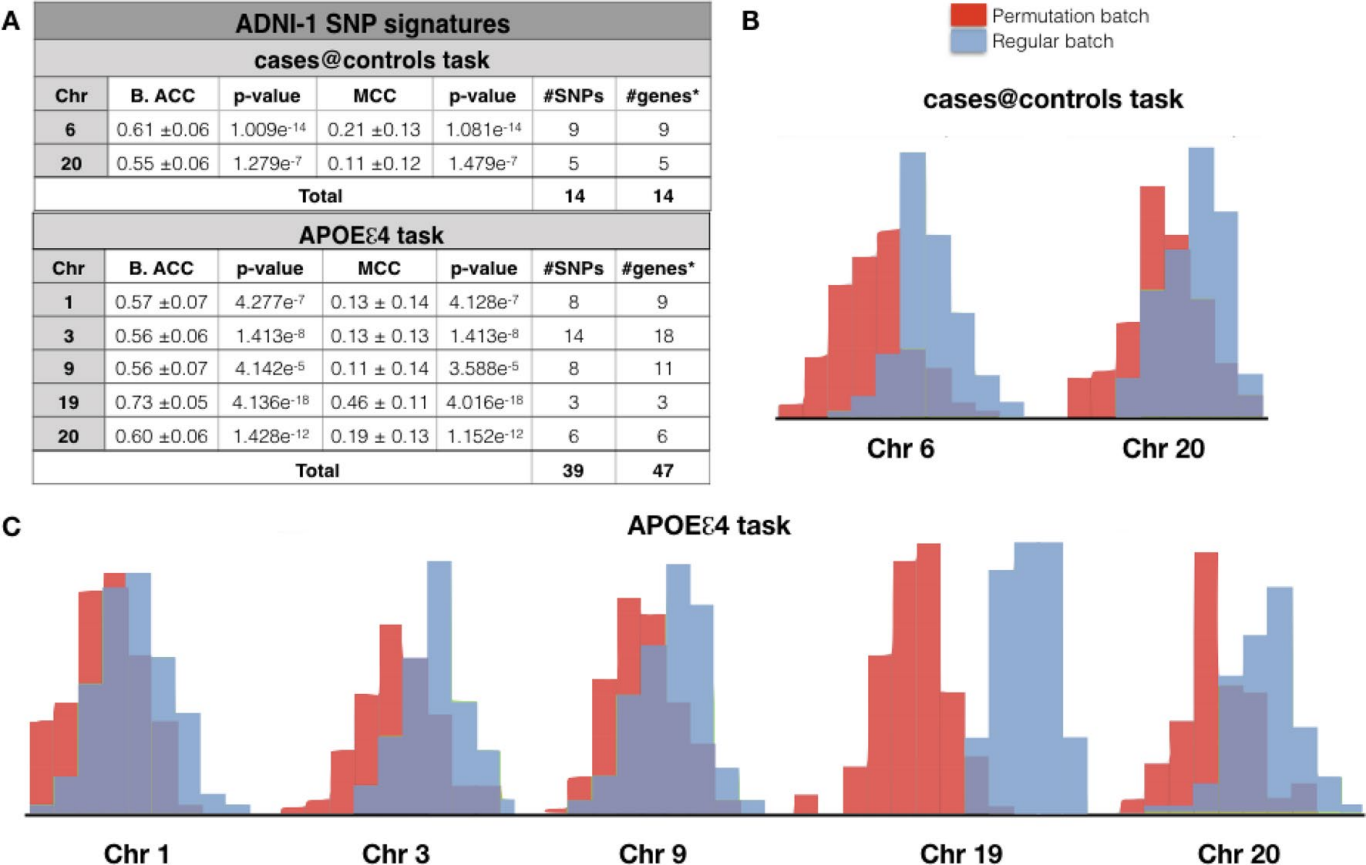
SNP-based results of ADNI-1. **(A)** The classification performance of SNP based analyses performed in ADNI-1 considering two classification tasks: AD vs. healthy controls (cases@controls) or 1/2 *APOEe4* vs. 0 *APOEe4* carriers (APOEe4 task). B. ACC, Balanced Accuracy; MCC, Matthews Correlation Coefficient; #genes*, number of genes or intergenic regions**. (B**) Balanced accuracy distribution plots of the regular (light blue) and the permutation batches (red) related to chromosomes 6 and 20 in the cases@controls task. **(C**) Balanced Accuracy distribution plots of the regular (light blue) and the permutation (red) batches related to chromosomes 1, 3, 9, 19 and 20 in the APOEe4 task.

The SNPs analysis performed on unimputed ADNI-1 dataset (Table S1) identified a signature of 14 SNPs relevant for cases@controls task (Fig. 2 and Table S2). These SNPs, mapped on 14 genes or intergenic regions and are located on chromosomes 6 and 20. In particular, chromosome 6 showed higher performance values with respect to chromosome 20, considering both balanced accuracy and MCC (0.61 ± 0.06 and 0.21 ± 0.13) (Fig. 2A). In addition, the higher distance between the regular (light blue) and the permutation (red) distributions of the calculated balanced accuracies, reinforced the robustness of the obtained results (Fig. 2B). Among the genes of this short SNP-signature, only *CDKAL1* is known to be associated to AD based on the literature^8^.

It is well recognized that *APOE* polymorphic alleles are the main genetic determinants of AD risk, being the individuals carrying one or two e4 alleles at higher risk to develop AD^3^. Considering that *APOEe4* polymorphism was harbored in 120 AD of 179 and 58 Control of 214, a further analysis based on the binary classification 1 or 2 *APOEe4* vs 0 *APOEe4* presence (APOEe4 task) was performed in order to characterize a polygenic profile that could uncover small effect size gene variants associated with the disease in a cumulative manner. 39 SNPs, which map to 47 genes or intergenic regions, have been identified in the APOEe4 task (Fig. 2A and Table S2). Chromosomes 19 and 20 were associated with the highest balanced accuracy and MCC results (Fig. 2A) and the distribution plots underlines this result (Fig. 2C). Based on the literature, 9 genes (i.e., red genes in Table S2) over a total of 47 are known to be involved in AD.

Interestingly, the two classification tasks (cases@controls and APOEe4 task) had in common *SHLD1* gene on chromosome 20, involved in the DNA double-strand breaks (DSBs) repairing mechanisms^9^. This gene is the closest to different SNPs found discriminant in the two tasks: in cases@controls task rs6053572 is located in the intergenic region between *GPCPD1* and *SHLD1* genes while in the APOEe4 task rs236137 and rs1287032 are located in the intergenic region between *SHLD1* and *CHGB* (Table S2).

**Figure 3:**
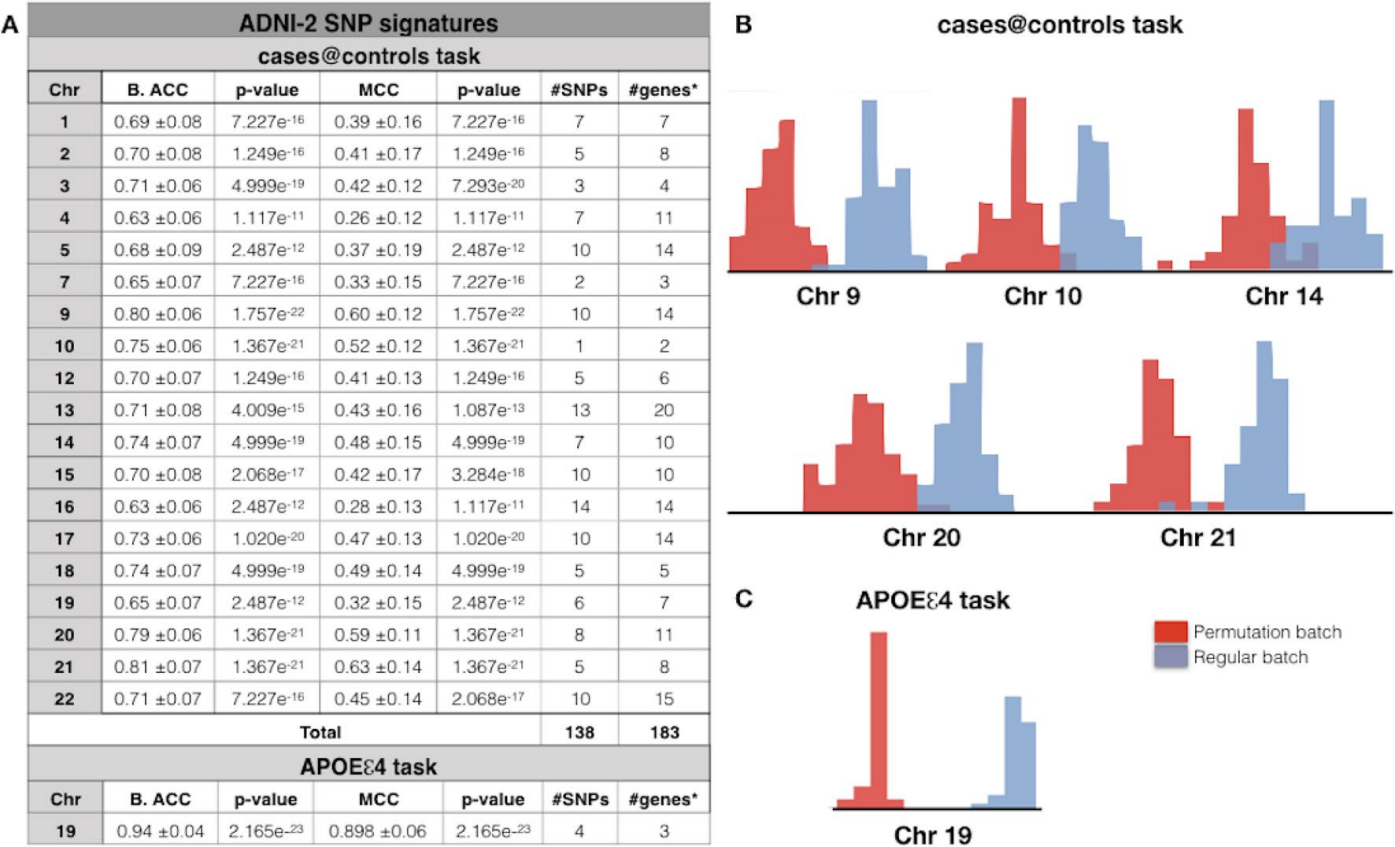
SNP-based results of ADNI-2. **(A)** The classification performance of SNP based analyses performed in ADNI-2 considering two classification tasks: AD vs. healthy controls (cases@controls) or 1/2 *APOEe4* vs. 0 *APOEe4* carriers (APOEe4 task). B. ACC, Balanced Accuracy; MCC, Matthews Correlation Coefficient; #genes*, number of genes or intergenic regions**. (B**) Balanced accuracy distribution plots of regular (light blue) and permutation (red) batches related to chromosomes 9, 10, 14, 20, 21 in the cases@controls task. **(C**) Balanced accuracy distribution plots of regular (light blue) and permutation (red) batches related to chromosome 19 in the APOEe4 task.

SNP-based analysis on unimputed ADNI-2 dataset (Table S1) identified for cases@controls task a signature of 138 SNPs, which map to 183 genes or intergenic regions harbored on 19 different chromosomes, with a balanced accuracy and MCC values ranging from 0.63 to 0.81 and 0.26 to 0.63 respectively (Fig. 3A and Table S3). In particular, chromosomes 9, 10, 14, 20 and 21 are the most reliable since they showed a higher distance between the two distribution measures (Fig. 3B). Based on the literature 12 genes (i.e., red genes in Table S3) over 138 are already known to be involved in AD.

When we considered the APOEe4 task, only chromosome 19 was found statistically significant, with very high values of both balanced accuracy (0.94) and MCC (0.90) (Fig. 3A and Table S3) and with very high distance between the two distributions (Fig. 3B). The derived SNP signature harbored only four SNPs located in three genes: rs367209 in *LOC101928063*, rs383133 in *ZNF221*, rs415499 in *ZNF155* and rs365745 that causes a missense mutation in *ZNF221* gene. Interestingly, *LOC101928063* and its rs367209 SNP, was found statistically relevant also considering ADNI-2-cases@controls task. Although none of these genes are already known to be associated to AD, according to the AlzGene database ^10^ (https://www.alzgene.org/), these SNPs are located in a linkage region (i.e.*, 19q13*) known to be associated to AD.

Considering SNP signatures of ADNI-1 and ADNI-2, we identified as a common gene *GRM7*, encoded for metabotropic glutamate receptor 7. ADNI-2 cases@controls task identified the intergenic SNP rs266410 between *MRPS35P* and *GRM7-AS3* (the antisense version of *GRM7*), while ADNI-1 APOEe4 task the SNP rs9311976, located in an intron inside *GRM7*.

### Gene-based results

In order to identify a gene signature for cases@controls and APOEe4 tasks, both the uninputed ADNI-1 and ADNI-2 datasets were analyzed by using three different tests included in the SKAT software (see Supplementary Information), applying a very conservative threshold for selecting a list of genes and SNPs highly relevant for AD (see Supplementary Information). In ADNI-1 dataset, *TOMM40*, with rs2075650, was found significantly associated to AD, applying all the tests (Table 1); while *TEF* gene, and in particular rs738499, was found significant in distinguishing cases@controls only with SKAT test (Table 1).

**Table 1:** Gene-based signatures identified in ADNI-1.

Gene symbol	P-value	Chr	Significant SNPs/# tot. SNPs
**Cases@controls task**			
*TOMM40*	2.21e^−7^	19	rs2075650/3 (intron)
*TEF*	9.97e^−6^	22	rs738499 (intron)/(2 intron + 1 coding)
*SAMM50|PARVB*	1.61e^−5^	22	rs2073080/3 (intergenic)
*BZW1*	1.82e^−5^	2	rs2270280 (intron)/4 (2 UTR + 2 intron)
*ZBED5|GALNTL4*	7.34e^−5^	11	rs12279328/121 (intergenic)
**APOEe4 task**			
*TOMM40*	7.88e^−38^	19	rs2075650/3 (intron)
*LOC100129500|APOC1*	9.64e^−16^	19	rs439401/2 (intergenic)
*TOMM40|APOE*	1.13e^−7^	19	rs405509/1 (intergenic)
*KPNA3|LOC2204429*	4.13e^−5^	13	rs11841624/24 (intergenic)

Lists of genes identified by the SKAT software in the cases@controls and APOEe4 tasks. The genes with P value < 1.37 × 10^–6^ are considered significant.

When the ADNI-1 dataset was analyzed considering the APOEe4,task, the genes or intergenic regions found significantly associated with AD risk were *TOMM40*, the intergenic region between *LOC100129500* and *APOC1*, and the intergenic region between *TOMM40* and *APOE* (Table 2). In particular, the two SNPs, rs439401 and rs405509 found in the intergenic regions *LOC100129500-APOC1* and in *TOMM40-APOE* respectively, were confirmed by all the SKAT three tests applied.

**Table 2:** Pathway-based signatures identified in ADNI-1 and ADNI-2.

	Dataset	Test score	Pathways signatures
ADNI-1 APOEε4 task	**Group 5a**	0.68	Amyloid fib. F., gamma carboxylation, unfolded protein resp., chaperonin, post-chap., **mitochondrial prot. import**., asparagine N-linked g., carboxyterminal post-t
	**Group 1c**	0.62	PIP3 activates AKT signaling, **detoxification of reactive oxygen species**
	**Group 9a**	0.62	**GPCR ligand b.,** pre-NOTCH expr. & proc., sign. by NOTCH3, sign. by NOTCH4
ADNI-2 cases@controls task	**Group 1c**	0.71	**Cellular senescence**, **detoxification of reactive oxygen species**
	Group 9c	0.67	Signaling by receptor tyrosine kinases
ADNI-2 APOEe4 task	Group 9c	0.71	Signaling by TGF-beta family memebers
	**Group 5a**	0.65	Amyloid fib. F., reg. of insulin growth factor, gamma carboxylation, unfolded protein resp., post-chap., **mitochondrial protein import**, asparagin., carboxylation
	**Group 1c**	0.64	**Cellular senescence**, **detoxification of reactive oxygen species**
	**Group 9a**	0.62	**GPCR Iigand b.,** pre-NOTCH e. p., sign. by NOTCH1, NOTCH2, NOTCH3, NOTCH4

Lists of the groups of pathways found statistically significant in APOEe4 task for ADNI-1 and in both tasks (cases@controls and APOEe4) for ADNI-2. The groups 1c, 5a, 9a were in common with ADNI-1 and 2. The test score shows the classification performance of Group Lasso with overlap. See Tables S4 and S5 for the complete list of all the pathways analyzed inside each group in ADNI-1 and ADNI-2 dataset respectively.

In bold are highlighted those groups of pathways that are in common among the different analysis performed in ADNI-1 and ADNI-2 dataset.

It is noteworthy that the SNP rs2075650 harbored in the introns of *TOMM40* was found in both ANDI-1 classifications tasks and it is confirmed in the literature to be associated to AD^11^.

Considering ADNI-2 dataset, the gene-based analysis did not give any significant association.

### Pathway-based results

For the pathway-based analysis we considered REACTOME database^12^. In particular, we chose specific pathway groups (Tables S4 S5), whose relevance in neurodegenerative processes were well recognized ^13^. With the ADNI-1 cases@controls task, no groups reaching statistical significance were found. At variance, different pathway groups, associated with AD risk (APOEe4 task), achieved a good test score (Table 2). In the ADNI-2 dataset the pathway analysis reached a good statistical significance in both the classification tasks addressed (i.e., cases@controls and APOEe4 tasks). Group c1 showed pathways in common across both datasets and classification tasks. In particular, the “detoxification of reactive oxygen species” pathway was found in ADNI-1 APOEe4 task and in both ADNI-2 tasks. The two ADNI-2 tasks share the “cellular senescence” pathway. Although c1 group “PIP3 activates AKT signaling” pathway was found significant only in ADNI-1, it is noteworthy for its relevance in different intracellular processes, including neuronal survival, metabolism, and glucose uptake, (Manning and Cantley 2007), whose down regulation has been associated with neurodegenerative disorders^14^. In addition, the two pathways, “mitochondrial protein import” and “GPCR ligand binding”, belonging to the group 5a and 9a respectively, were identified in the APOEe4 tasks of both datasets (Table 2). Interestingly, these two latest pathways involved *TOMM40* and *GRM7* gene respectively, previously identified by SNPs and gene-based analysis.

In addition, we also performed a functional characterization in KEGG database (see Supplementary Information), in order to further biologically characterize the gene lists derived from the SNPs signatures identified before. A successful analysis was obtained only for ADNI-1 APOEe4 task and ADNI-2 cases@controls task, whose SNP signatures reported a long SNPs’ list (Table 3). In ADNI-1 APOEe4 task only the “Neuroactive ligand-receptor interaction” pathway, that includes the genes *P2RY13*, *GRIN3A*, *LEPR*, *GRM7*, *P2RY14*, reached a significant adjusted P value (Adj-P value = 5.99e−06).

**Table 3:** Functional characterization in KEGG.

	Functional characterization			
	KEGG database			
	Pathway name	#Genes	Adj-P-value	Gene symbol
ADNI-1	**Neuroactive ligand-receptor interaction**	5	5.99e-06	*P2RY13, GRIN3A, LEPR, GRM7, P2RY14*
	**Pathway name**	**#Genes**	**Adj-P-value**	**Gene Symbol**
ADNI-2	Calcium signaling pathway	4	0.0040	*PLCB4, GRIN2A, CYSLTR2, ADCY9*
	**Neuroactive ligand-receptor interaction**	4	0.0064	***GRM7, GRIN2A, CYSLTR2, GABRG3***
	Axon guidance	3	0.0064	*ABL1, EPHA6, NTN1*
	Chemokine signaling pathway	5	0.0080	*PLCB4, GNG2, JAK2, NFKB1, ADCY9*

Pathways enrichment results of the SNPs signatures identified in ADNI-1 and ADNI-2 dataset, considering the APOEe4 and cases@controls tasks respectively. #genes*, number of genes Adj-P-value, adjusted P-value.

The pathways names highlighted in bold are commented in the main text.

In ADNI-2 the most important pathways related to AD were “Chemokine signaling”, “Calcium Signaling”, “Axon Guidance” and “Neuroactive ligand-receptor interaction” (Table 3). The latter pathway, in common with ADNI-1, involved *GRIN2A*, *GRM7*, *GABRG3* and *CYSLTR2* genes.

## Discussion

Despite the promise of GWAS to reveal the genetic contribution to AD susceptibility, the majority of its heritable component remains unexplained. The major factor contributing to hamper the identification of genetic burden lies in the complexity of GWASs data management, together with the genetic heterogeneity of AD. In fact, although GWAS studies have revealed a plethora of putative susceptibility genes for AD, *APOE* gene is the sole exception unequivocally validated in independent studies.

The final purpose of the alternative strategy that we present in this study is to contribute in uncovering a robust heritable AD signature in the analysis of GWAS data. The key points of this strategy are the following: a new representation of the genotyped SNPs data; the telescope approach, since the data were analyzed at SNPs, genes and pathways levels; the choice of multivariate machine learning methods ad hoc for the three levels; the analysis of two separate dataset, each addressing two relevant binary classification tasks: cases@controls and APOEe4.

The new data representation have improved the classification performances of the applied machine learning algorithms because (i) it changed the nature of the data (from categorical to continuous), improving their interpretability for the considered machine learning methods and (ii) it made a more evident and biologically sound prioritization of some SNPs with respects to others. The analysis at SNPs, genes and pathway levels allowed the comparison of the results at these three levels and the successive identification of common related features, increasing their robustness in the association with AD. We chose the most appropriate machine learning method based on the characteristics of the analyzed data: l_1_l_2FS_ was used to analyzed SNPs data because it is a sparse method, meaning that the solution to the classification problem is searched among a precise selection of the most relevant SNPs. This feature is essential to discard all those SNPs that are background noise or that are weakly associated to the addressed classification problems. The precise choice of the algorithm together with the SNP data transformation improved the selection of the most relevant SNPs from both the statistical and biological viewpoints. For the analysis of the gene level we select SKAT method because it provides the user the possibility to weight the SNPs differently based on their frequency occurrence in the subjects of the SNP dataset. Group Lasso with overlap was chosen because it is characterized by a feature that we seek in the analysis of the pathways: while looking for the most discriminant pathways within a group of them, which in this work constitute a single SNPs data matrix, we wanted the algorithm to consider the involvement of a gene in more then one pathway of the group. Finally the motivation behind the choice of addressing two classification tasks and the separate analysis of ADNI-1 and ADNI-2 dataset is to compare the results, between different biological questions and between two independent studies respectively. Besides the cases@controls classification task, where the disease is the discriminant, we considered also the APOEe4 task because studies have shown that individuals with two copies of the e4 allele are at even greater AD risk, and the odds ratios for developing AD based on *APOE* is 5 times greater in *APOEe4* homozygotes compared to heterozygotes^15^. Therefore, a binary classification based on the presence of almost one *APOEe4* allele could (i) uncover a cumulative polymorphic risk variants contributing to AD predisposition, and/or (ii) highlight superimposable genetic fingerprint, allowing a better understanding of *APOE* genotype contribution in the disease etiology. In addition, this classification might give useful insights for better addressing the therapeutic strategies, since multiple studies over the past two decades have demonstrated that *APOE* variants may affect the therapeutic response to anti-dementia drugs^16–19^*.* In this context, very recently, Berkowitz et al. ^15^ claimed that in the prospective of clinical precision strategy, the APOEe4 carrier status could have a very important impact on AD prevention interventions.

Considering the single signatures and datasets, in this study we identified lists of SNPs and genes, some of which are already reported in literature (see red colored SNPs and genes in Tables S2 and S3). But, in the tentative to adopted highly stringent and powerful statistical correction (permutation-regular batch for SNPs analysis and genome wide conservative threshold for genes analysis) to avoid false-positive results and increase the robustness of AD signatures, the numbers of SNPs and genes associated with AD or AD risk is strongly reduced. Even, the gene analysis of ADNI-2 did not give any results.

Furthermore, when we compared the two ADNIs datasets the majority of the signatures identified in ADNI-1 were not confirmed in ADNI-2 for both classifications tasks, although the demographic and clinical characteristics of the subjects enrolled in the two studies were comparable. A possible explanation of the low reproducibility of the results between the two datasets could be due to the following issues: (i) the different Illumina GWAS platforms, (ii), the lack of imputing procedure before the independent analysis of the two dataset (iii) and the difference in the genotype of APOE gene between ADNI-1 and ADNI-2 datasets. ADNI-1 and ADNI-2 datasets measured 620,901 and 730,523 SNPs respectively, of which only 300,000 were in common. An imputation procedure of ADNI-1 and ADNI-2 could have increased the SNPs overlap between the dataset and therefore could have increased the number of relevant SNPs genes and pathways and in turn allowed the validation of all the SNPs signatures identified in ADNI-1 and ADNI-2.

In addition, the fact that in ADNI-1 dataset *APO*E gene was genotyped separately from the other genes present in the platform, differently from ADNI-2, had surely influenced the obtained results in the SNPs and genes analyses.

The only heritable susceptible gene confirmed across SNPs and genes in ADNI-1 and across pathways in both datasets was *TOMM40*. On the other hand, the SNP and the pathway analysis of both ADNI-1 and ADNI-2 uncovered *GRM7* gene as significantly associated with AD or AD susceptibility genetic profile.

*TOMM40* is located in *19q13.32* locus, a known linkage region for AD^10^. Its encoded protein plays a key role in the mitochondria functionality being essential for import of protein precursors into mitochondria. The SNPs-based analysis identified two SNPs rs2075650 and rs8106922 harbored on *TOMM40* gene, for ADNI-1 APOEe4 task. At the same time the SNP rs2075650 located in one intron of *TOMM40*, has been identified by the genes analysis of both controls@cases and APOEe4 task of ADNI-1 dataset. The literature confirms that *TOMM40* gene is deeply involved in AD pathology^20–22^, and in particular that rs2075650 SNP is already known to be a contributing factor for AD (Huang et al*.* 2016; Potkin et al*.* 2009).

*TOMM40* was also confirmed by pathway analysis in both dataset for the APOEe4 task. In particular, the protein encoded by *TOMM40* is involved in the “mitochondrial protein import” pathway**.** The fact that SNPs, genes and pathways analyses highlighted the strong association between *TOMM40* variants and *APOEe4* genotype is due to *TOMM40* location in the tight gene cluster *TOMM40-APOE-APOC1-APOC4-APOC2* that is a strong linkage disequilibrium (LD) block ^25^. Furthermore, it has been reported that the *APOE-TOMM40* genomic region has been associated with cognitive aging^26^ and with pathological cognitive decline^27^. *APOE-TOMM40* genotypes have been also shown to modify disease risk and age at onset of symptoms^28,29^.

Interestingly, although ADNI-2 showed a long list of susceptible genes in SNPs analysis, *TOMM40* did not emerge, probably due to the lack of datasets imputation. On the other hand, in ADNI-2 APOEe4 task, *TOMM40* appeared in the pathway “mitochondrial protein import”.

*GRM7* represents a novel possible candidate gene that needs to be experimentally validated, for the association with AD. It is located in *3p26.1* locus, and encodes for the metabotropic glutamate receptors 7, involved in the presynaptic neurotransmitter regulation^30^. *GRM7* was identified in the SNPs and pathways analyses of both ADNI-1 and ADNI-2 dataset. In particular, the SNP rs9311976 on *GRM7* gene was found in ADNI-1 APOEe4 task, while rs266410 in the inter region *MRPS35P1/GRM7-AS3* (the antisense of *GRM7*) of ADNI-2 controls@cases task. *GRM7* was also identified in “GPCR ligand binding” pathway, belonging to the REACTOME group 9a in the APOEe4 tasks of both datasets (Table 2), and confirmed by the in silico functional characterization that found enriched the same KEGG pathway “neuroactive ligand-receptor interaction” in both ADNI-1 and ADNI-2 SNP signatures. The identification of *GRM7* gene in the APOEe4 classification task corroborated the association of glutamate signaling with *APOE* genotype. In fact, reduced expression of glutamate receptor proteins has been found in *APOEe4* carrier AD^31^ and a defective glutamate synthesis has been shown in presynaptic *APOEe4* neurons^32^. Furthermore, *GRM7* has been found involved in schizophrenia^33^ and other mental disorders^34,35^. These finding were also confirmed by epidemiologic studies that showed significant associations between *GRM7* and depression, anxiety, schizophrenia, bipolar disorder, and epilepsy^36,37^. Recently it has also been demonstrated that 3xTg-AD mice showed lower *GRM7* protein expression in hippocampus, associated with an increased anxiety behavior, compared with the wild-type mice^38^. The significance of such results were confirmed by a genome-wide gene and pathway-based analyses on depressive symptom burden in the three independent cohort derived from the Alzheimer's Disease Neuroimaging Initiative (ADNI), the Health and Retirement Study (HRS), and the Indiana Memory and Aging Study (IMAS)^39^. In addition, *GRM7* has been confirmed associated with AD in a meta-analysis of GWAS studies where glutamate signaling genes were found overrepresented in KEGG pathway enrichment analysis^40^.

In conclusion, the alternative GWAS analysis strategy applied in the analysis of two unimputed ADNI datasets, identified *TOMM40* and *GRM7* polymorphic variants as strongly associated with AD. Their relevance was confirmed by the identification of the mitochondrial import and glutamatergic signaling pathways, identified by pathway analysis, in which *TOMM40* and *GRM7* are respectively involved. Furthermore, the fact that these genes were found strongly associated with *APOEe4* status at the SNPs, genes and pathway levels, corroborated its significance in the context of a cumulative polygenetic susceptibility to AD.

Although a possible limitation of this work could be found in the preprocessing phase and specifically in the absence of the imputation step, that could had improved the overlap of ANDI-1 and ADNI-2 dataset and the validation of the identified SNPs signatures, we strongly believe that this alternative approach of GWAS analysis presented in this study could provide a valuable way to uncover the genetic hereditability of multifactorial diseases like AD. In the future we plan to (i) test our approach applying the imputation step before the reanalysis of ADNI-1 and ADNI-2; (ii) to validate the results obtain in ADNI-1 in ADNI-2 and viceversa, following the strategy adopted in this work; (iii) to validate the identified SNPs, Genes and Pathways signatures in other independent GWAS dataset; (iv) to integrate covariates data, such as clinical characteristics of the patients to the analysis of the most discriminant SNPs, Genes and Pathways identified before.

## Material and methods

### Datasets

Data used in the preparation of this article were obtained from the Alzheimer’s Disease Neuroimaging Initiative (ADNI) database (https://adni.loni.usc.edu/). The primary goal of ADNI has been to test whether serial magnetic resonance imaging (MRI), positron emission tomography (PET), other biological markers, and clinical and neuropsychological assessment can be combined to measure the progression of mild cognitive impairment (MCI) and early AD. In this study GWAS data and *APOE* genotype obtained in the ADNI-1 and ADNI-2 datasets ^41^ were used (Table S1), considering the AD and healthy controls (CN) group. The genotyping platforms used by ADNI-1 and ADNI-2 were: Illumina Human 610-Quad BeadChip that measures 620.901 SNPs and CNV markers for ADNI-1 and Illumina Human OminExpress-24v that measured 730.525 SNPs and CNV markers for ADNI-2. Differently from ADNI-2, in ADNI-1 *APOE* genotyping is provided outside the GWAS platform. In both datasets, we performed two supervised binary classification analyses: AD vs. cognitively healthy subjects (cases@controls task) and subjects at higher risk vs. subjects not at risk of developing AD, according with *APOE* status (1 or 2 alleles vs. 0 allele of *APOEe4*) (APOEe4 task) (Table S1). Since the two dataset have been generated by two different Illumina platforms, showing a discrepancy between the number of SNPs and the APOE genotype presence, they were analyzed separately without performing the imputation step before the analysis phase. In addition the validation of ADNI-1 signatures in ADNI-2 dataset and of ADNI-2 signatures in ADNI-1 dataset has also been performed (see Supplemental Results).

### Alternative GWAS analysis strategy

An alternative approach was devised to analyze the datasets. Considering the two classification tasks addressed and the SNPs, Genes and Pathways levels, each ADNI dataset was analyzed six times (Fig. 1).

In order to increase the signal over noise ratio, reducing the number of SNPs to analyze, we adopted the following strategy: (1) for the SNP and pathway analyses we employed two sparse methods (i.e.*,* l_1_l_2FS_ and Group Lasso with overlap), designed to identify the SNPs or pathways which are most discriminative for the classification tasks while restricting the selection of SNPs and pathways, and we considered a different representation of the SNP data (see Supplementary Information); (2) for the SNP analysis we analyzed each chromosome separately while for the gene and pathway analyses we grouped the SNPs considering genes/intergenic regions and pathways relevant for AD respectively.

### SNP analysis

For the SNP analysis, we chose l_1_l_2FS_, a method that belong to sparse techniques^42^. This method allows the identification of the most discriminative variables for the problem at hand (classification tasks) while making feature selection (see Supplementary Information). l_1_l_2FS_ was used within PALLADIO^43^ (https://slipguru.github.io/palladio/), a machine learning framework that can be customized to consider various combinations of feature selections and classification methods (Figure S1A). In order to ensure the reliability of the results, we used PALLADIO to perform two sets of experiments, which we referred to as *regular* batch and *permutation* batch (Figure S1B). The level of distance of the two distributions measured the reliability of the obtained results: the higher the distance, more reliable are the obtained results (see Supplementary Information).

### Gene analysis

For the gene analysis, we considered three association tests available in the SKAT package (https://www.hsph.harvard.edu/skat/): Burden, SKAT and SKATO (see Supplementary Information). SKAT^44,45^ is a supervised regression method that test the association between genetic variants in a region and a dichotomous or a continuous trait while adjusting for covariates. The dichotomous traits considered here were cases@controls and APOEe4 tasks. In the first application of this alternative GWAS analysis strategy we chose to exclude covariates such as age at onset, race, sex. Furthermore we chose to consider genes or intergenic regions leveraging on the mapping files SNPs-to-genes provided by the GWAS platform manufacturer (i.e., “Human610_Gene_Annotation_hg19.txt” for ADNI-1 and “HumanOmniExpress-24v1-1_Annotated.txt” for ADNI-2).

The threshold of genome-wide significance we established, was conservative and in accordance with other studies^46–49^ (see Supplementary Information).

### Pathway analysis

We selected 9 groups of pathways more relevant for neurodegenerative processes (Tables S4 and S5) inside REACTOME database^50^(https://reactome.org/).

In this study the pathways we selected and analyzed are the same for both dataset but some groups can differs in the number of pathways between the datasets because there is a low overlap of analyzed SNPs between the used platforms of ADNI-1 and ADNI-2 dataset.

Each group contained two or more pathways and each group represented a SNP matrix that, together with a label that characterizes each subject, was given as input to “Group Lasso with overlap”^42^. This latter is a machine learning method, able to consider the presence of overlapping groups of SNPs mapped to genes, involved in more than one pathway inside a group. The goal of “Group Lasso with overlap” is to induce a “sparse” selection at the group level, using all the pathways specified in the group. In this way, starting from a possibly long list of pathways inside a group, the algorithm selected a few (but informative) pathways that could be relevant for the problem at hand.

## Supplementary information

Supplementary Information.
