# Supplementary material for: A telescope GWAS analysis strategy, based on SNPs-genes-pathways ensamble and on multivariate algorithms, to characterize late onset Alzheimer’s disease
Source: Sci Rep. 2020 Jul 21;10:12063. doi: 10.1038/s41598-020-67699-8 (PMC7374579; doi:10.1038/s41598-020-67699-8)
Supplement: Supplementary file 1 — Supplementary Information. [file 41598_2020_67699_MOESM1_ESM.pdf]

Supplementary Information

A telescope GWAS analysis strategy, based on SNPs-Genes-Pathways ensamble and on multivariate algorithms, to characterize Late Onset Alzheimer's Disease

Margherita Squillario[1,*], Giulia Abate[2], Federico Tomasi[1], Veronica Tozzo[1], Annalisa Barla[1] and Daniela Uberti[2] "for the Alzheimer's Disease Neuroimaging Initiative"

* **Correspondence**: margherita.squillario@unige.it

Method Details

Data matrix transformation

Machine learning methods are state-of-the-art methods for a number of high-throughput data but they are still not very popular in the field of SNP analysis. Two are the main reasons: first, SNP data are usually very high-dimensional making some algorithms difficult to use on a regular workstation and second SNPs are usually represented by categorical variables (0, 1 or 2), a data type which may lead to inconsistent results with some machine learning methods.

Therefore for the SNP and pathway analyses we decided to use a different representation of the data that we defined "reinforced" and that uses weights calculated with a function of the Sequence Kernel Association Test (SKAT) method (see Supplementary Information), employed in the gene analysis performed in this work. Specifically we divided the samples of each datamatrix in two equal halves: one of these halves was used to calculate a weight for each SNP and the other half was used to address the actual classification task. Before this latter analysis we modified the data matrix multiplying the usual additive *code* associated to each SNP (*i.e.,* 0,1, or 2) to its respective weight, defined *beta,* as shown in this formula:

$$SNP_{(i)} = SNP_{code(i)} * beta_{(i)}$$

The few missing values present in the data matrix were replaced with the weight value associated to that SNP. Using this procedure we obtained datamatrices of continuous values, more suitable to be analyzed by many machine learning methods.

The SKAT function used to calculate these SNPs weights is called "Get Logistic Weights" and its formula is:

$$weights = \frac{e\hat{}(par1 - MAF)par2}{1 + e\hat{}(par1 - MAF)par2}$$

The SNPs weights are obtained considering the minor allele frequencies (MAF) that refer to the frequency at which the second most common allele occurs in a given population. It is an index used in population genetics to distinguish between common and rare variants. *Par1* and *par2* are two parameters, for the common and rare variants respectively, that a user can set up in order to give more or less relevance to rare and common variants. In this study we used the values suggest in [1] in order to give a value different from 0 but very low (*i.e.,* par1 = 1) to common variants and an high value to rare variants (*i.e.,* par1 = 25), which are thought to have a major role in complex diseases like AD.

SNP analysis

When dealing with high-dimensional data, a natural problem arises in relation to the low number of available samples *n* with respect to the dimensionality of the problem that concerns the number of features *p* (*i.e., n << p*). This issue happens frequently when dealing with biological data, and it usually referred to as "curse of dimensionality". In this setting, usual statistical guarantees are lost, since the problem is over-determined, *i.e.,* there is no unique solution to the problem. A way to overcome this difficulty is to incorporate prior knowledge into the problem at hand, for example by employing sparse techniques, such as Elastic-Net or l_1l_2 feature selection (l_1l_{2FS}) [2].

l_1l_{2FS} validate the robustness of the method using a model assessment framework, for which the model we selected as the "best" on our data is trained on a portion of the data (*learning set*) and tested on other unseen data (*test set*), iteratively (Figure S1A). Following this procedure it is possible to obtain a performance score for each selected model, through which we ensure the generalization properties our model achieves on unseen data.

In the present work we chose l_1l_{2FS} within PALLADIO [3] a machine learning python framework that can be customized to consider various combinations of feature selections and classification methods. Independently of the chosen methods, this tool ensures the reliability of the results performing two sets of experiments, *regular* and *permutation* batches (Figure S1B).

For each experiment, we resamples different learning and test sets a large number of times, in order to estimate the performance score distribution for both batches. The regular batch performs experiments on the given dataset, while the permutation batch performs experiments where the relationship between input and output is destroyed by shuffling the labels in the learning set, following what is referred to as *permutation test*. The two distributions are then compared by testing the null hypothesis H0 by means of a Two-sample Kolmogorov–Smirnov test [4], a principled way to measure the statistical robustness of the obtained result. Then, we can reject the null hypothesis when the computed p-value is smaller than the confidence interval. Rejecting H0 implies a clear difference between the two distributions and the sample size is large enough to describe the relationship between data and labels. The final outcome of a classification process is the prediction of the labels associated with a set of input samples. In order to assess the performance of l_1l_{2FS}, PALLADIO computes, among other performance metrics, the *balanced accuracy score* and the *Matthews correlation coefficient* (MCC).
The balanced accuracy score is the ratio of correctly predicted labels, adjusted for unbalanced problems. In particular, a predictor that always returns the label of the most represented class would yield a score of 50%, independently of the proportion of the labels in the dataset. MCC, also, is a comprehensive measure shown to be particularly useful for unbalanced problems, since it is always defined to be +1 for perfect match between predicted labels and ground truth, −1 for total disagreement and 0 for random prediction.

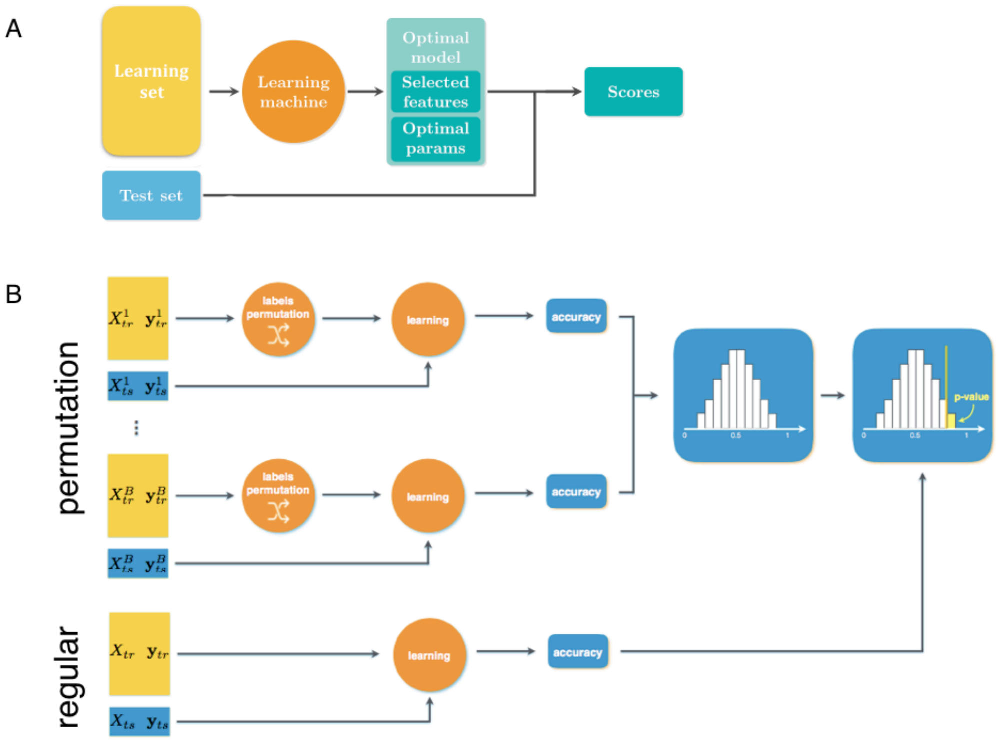

Figure S1. l1l2 and PALLADIO schemes.

(A) It shows the model assessment framework of l_1l_{2FS}.

(B) It shows the approach that PALLADIO adopts to ensure the reliability of the results.

Gene analysis

SKAT [1] uses a multiple regression model to directly regress the phenotype on genetic variants in a region and on covariates allowing different variants to have different directions (i.e., protective or causal) and magnitude of effects, including no effects. To collapse the information of the

variants in a region, SKAT does not need a threshold because it uses a variance-component score test that is a kernel association test. In the formula of this test there are those weights that a SKAT user can chose to improve the power of the analysis (see "Data matrix transformation" section in Supplementary Information).
In the present work we chose to utilize the *SKATBinary* package, more suitable with PLINK formatted files, that encloses a function that computes p-values for Burden, SKAT and SKAT-O test for binary traits (in our case cases@controls and APOEe4 tasks) using asymptotic and resampling methods.
Burden test is suitable in case when a large proportion of variants are causals and their effect are in the same direction (*i.e.,* all protective or causal). SKAT test is suitable when only a small proportion of variants are causal or their effects have mixed directions (*i.e.,* some protective and some causal). SKAT-O test is suitable whenever we have a genetic scenario that is a mixture between that one suitable for Burden and that one suitable for SKAT.
For ADNI-1 and ADNI-2 we applied the following conservative thresholds: 0.05/36,000 = 1.37×10^{-6} and 0.05/29.484 = 1.70×10^{-6}, where 0.05 is the level of significance, and the denominator indicates the known genes and intergenic regions in which the platforms have been subdivided.

In silico SNP characterization

The functional characterization of the gene lists derived from the SNPs signatures identified with PALLADIO was performed through enrichment analysis using the online toolkit WebGestalt [5]. This tool takes as input a list of relevant genes/probesets and performs an enrichment analysis based on a hypergeometric test, providing several methods to correct for multiple hypothesis and using several databases (*e.g.,* the Kyoto Encyclopedia of Genes and Genomes (KEGG), Gene Ontology (GO)) for identifying the most relevant pathways and ontologies in each signature. In other words, given a KEGG pathway and a reference set (such as the entire human genome or the list of genes in a microarray platform), the enrichment is based on the comparison between the fraction of lists genes in the pathway and the fraction of KEGG pathway genes in the reference set. The gene list is enriched in that specific KEGG pathway if the former is larger than the latter fraction.
In the present work we enriched the two longest gene lists deriving from the SNP signatures of ADNI-1 (APOEe4 task), and of ADNI-2 (cases@controls task) considering KEGG [6] a database of pathway. In this analysis we considered the human genome as reference, 0.05 as level of significance, Benjamini-Hochberg as test for multiple hypothesis correction and 3 as the minimum number of genes in a KEGG pathway.
In order to understand which genes of the identified SNP signatures are already known to be involved in AD and which genes are not, we utilized Phenopedia [7], two web-based applications that explore the literature in a gene-centric and disease-centric way. We obtained the list of genes associated to AD from Phenopedia and we compared it to our lists of genes derived from our SNP signatures. The genes highlighted in red in Tables S2 and S3 are those genes known to be involved in AD.

Supplementary tables

	**ADNI-1**	**ADNI-2**
**AD cases**	179	126
**Controls**	214	155
**1/2 APOEe4 carriers**	178	120
**0 APOEe4 carriers**	215	161

Table S1. Sample size of the classes compared in the two classification tasks addressed in ADNI-1 and ADNI-2 datasets. AD cases vs. controls define the cases@controls task while 1/2 APOEe4 carriers (high risk) vs. 0 APOEe4 carriers (low risk) define the APOEe4 task.

	**ADNI-1**		
	**SNP**	**# Chr**	**Gene Symbol**
**Cases@Controls**	rs12205042	6	*HIVEP2*
	rs1940890	6	*LOC101928911 | SPACA1*
	rs16881241	6	*RNGTT | LOC100131124*
	rs6914160	6	*KLHL31 | LRRC1*
	rs9465982	6	***CDKAL1***
	rs543049	6	*LOC101928911 | SPACA1*
	rs6923298	6	*HIVEP2*
	rs677120	6	*LOC101928911 | SPACA1*
	rs4896228	6	*IL20RA*
	**rs6053572**	**20**	***GPCPD1 | SHLD1***
	rs708925	20	*PLCB1*
	rs2983626	20	*CST9L | CST9*
	rs2247337	20	*CST9L*
	rs13040567	20	*CST9L*
**APOEe4**	rs11260977	1	*IGSF21*
	rs4839223	1	*SLC6A17*
	rs4839225	1	*SLC6A17*
	rs10749753	1	*LEPROT|**LEPR***
	rs1338138	1	*LINC01781|MTND2P30*
	rs11102933	1	***NGF**|LOC112268234*
	rs2147085	1	*LINC01781|MTND2P30*
	rs10789215	1	*SGIP1*
	rs12487324	3	*IQSEC1|NUP210*
	rs8180086	3	*GPR87|P2RY13*
	rs7651843	3	*RBMS3*
	rs7641352	3	*TBL1XR1|**KCNMB2***
	rs276117	3	*LOC728290|GBE1*
	rs9875152	3	***P2RY12**|MED12L*
	rs7625229	3	*BFSP2|LOC391578*
	rs661798	3	*KALRN*

	rs12491760	3	***PTPRG***
	rs9311976	3	***GRM7***
	rs9310917	3	*RBMS3*
	rs6784803	3	*MECOM*
	rs7653603	3	***P2RY14***
	rs276125	3	*LOC728290|GBE1*
	rs13283389	9	***GRIN3A***|*CYLC2*
	rs11139921	9	*RASEF|FRMD3*
	rs10820215	9	***GRIN3A***|*CYLC2*
	rs7046513	9	***GRIN3A***|*CYLC2*
	rs10119403	9	*LINC01505*
	rs1891999	9	*OLFM1|C9orf62*
	rs7041138	9	*SLC25A6P5|LINC01505*
	rs1350996	9	*FLJ35282|LOC101929563*
	**rs2075650**	**19**	***TOMM40***
	**rs8106922**	**19**	***TOMM40***
	**rs439401**	**19**	***LOC100129500|APOC1***
	**rs236137**	**20**	***SHDL1|CHGB***
	rs6041265	20	*BTBD3|PA2G4P2*
	**rs1287032**	**20**	***SHDL1|CHGB***
	rs6041271	20	*BTBD3| PA2G4P2*
	rs2294575	20	*CHD6*
	rs2057291	20	*GNAS*

Table S2. ADNI-1 SNP signatures identified in the cases@control and in the APOEε4 tasks. "# Chr" indicates the chromosome number and "|" in the Gene Symbol column indicates that a specific SNP is located in a intergenic region between two genes.
In red color are highlighted those genes that are already known to be associated to AD. In bold black color are highlighted those genes/SNPs mentioned in the main manuscript.

	**ADNI-2**		
	**SNP**	**# Chr**	**Gene Symbol**
**Cases@Controls**	rs640688	1	*HIVEP3*
	rs2093933	1	*KIAA1324*
	rs3913318	1	*MIR4471 | LOC100287877*
	rs10493973	1	*OLFM3*

	rs2843130	1	*MORN1*
	rs724309	1	*ATPAF1*
	rs6739882	2	*MIR4431 | ASB3*
	rs13014133	2	*RBMS1 | LOC100131736*
	rs7562244	2	*AGAP1*
	rs2160782	2	*LINC01800 | LINC02245*
	rs13389584	2	*GPR39*
	rs266410	3	*MRPS35P1 |* ***GRM7-AS3***
	rs341981	3	*EDEM1*
	rs1598915	3	*EPHA6*
	rs9683798	4	*ZEB2P1 | LDB2*
	rs7660498	4	*HAUS3 | MXD4*
	rs224489	4	***HS3ST1*** *| LOC101929019*
	rs4689726	4	***SORCS2***
	rs12507259	4	*STK32B*
	rs2203758	4	*LCORL | RPL21P46*
	rs4648016	4	***NFKB1***
	rs10043779	5	*KIAA0825*
	rs1494699	5	*MSNP1 | LOC100131678*
	rs6882967	5	*MSNP1 | LOC100131678*
	rs25754	5	*ADAMTS12*
	rs17156151	5	*NUDT12 | RAB9BP1*
	rs261747	5	*FYB*
	rs6892938	5	*ARL15*
	rs2028269	5	*MTX3 | LOC100500934*
	rs6865330	5	*FGF10-AS1 | LOC100506674*
	rs9647537	5	*PGBD3P2 | HPRTP2*
	rs17136076	7	*RNA5SP230 | MYL7*
	rs17166226	7	*SCIN*
	rs10121110	9	*ENG*
	rs9792690	9	*TRPM3*
	rs10976614	9	*C9orf123 | PTPRD*
	rs4740366	9	***ABL1***
	rs9408761	9	*PTPRD*
	rs7854386	9	*LOC401557 | C9orf62*
	rs36100013	9	*JAK2*
	rs10817547	9	*ZNF618*

	rs10819687	9	*NAMA | LOC101928438*
	rs7031871	9	*ARL2BPP7 | LOC100127962*
	s11244450	10	***CHST15*** *| OAT*
	rs4980929	12	*IQSEC3*
	rs11609462	12	*ERC1*
	rs3759347	12	***LEPREL2***
	rs3217933	12	*CCND2*
	rs4766200	12	*PARP11 | HSPA8P5*
	rs9552886	13	*SGCG*
	rs7996072	13	*CYSLTR2*
	rs17085790	13	*LNX2*
	rs12867878	13	*RNA5SP30 | LOC101926897*
	rs11841581	13	*TEX26 | WDR95P*
	rs10507296	13	*MIPEPP3|LINC00539*
	rs9564566	13	*SNRPFP3 | SRSF1P1*
	rs1373904	13	*LACC1 | DGKZP1*
	rs2389229	13	*ABCC4*
	rs7999070	13	*TPTE2P1*
	rs12861751	13	*LINC00378 | MIR3169*
	rs1935179	13	*RPL7L1P1 | PEX12P1*
	rs2407249	13	*CYSLTR2 | PSME2P2*
	rs12894732	14	*LOC100418768 LINC01800*
	rs10136784	14	*LINC00639*
	rs7143462	14	*ESRRB | CYCSP1*
	rs2748144	14	*LOC101927598 | GNG2*
	rs3825604	14	*GNG2*
	rs11156929	14	***SLC25A21***
	rs8010556	14	*C14orf132*
	rs6497287	15	*HERC2*
	rs2672680	15	*FAM189A1*
	rs870185	15	*ZFAND6*
	rs1883005	15	*SNORD115-21|SNORD115-15*
	rs12908255	15	*PSTPIP1*
	rs11634439	15	*ARHGAP11A*
	rs2010459	15	*TMED3*
	rs4965785	15	*LRRK1 | CHSY1*
	rs7167588	15	*GABRG3*

	rs34261044	15	*HERC2*
	rs2239307	16	*ADCY9*
	rs8061043	16	*CLEC16A*
	rs12598337	16	*GRIN2A | ATF7IP2*
	rs11643000	16	*GRIN2A*
	rs6497898	16	*HS3ST4*
	rs36474	16	*MYLK3*
	rs9933735	16	*RBFOX1*
	rs1124018	16	*RBFOX1 | LOC100131080*
	rs4390571	16	*RBFOX1 | LOC100131080*
	rs9940785	16	*RBFOX1*
	rs2075158	16	*RSL1D1*
	rs3116150	16	*SLC5A2*
	rs13330742	16	*WWOX*
	rs7189472	16	*XPO6*
	rs2079268	17	*ALOX15P1 | SLC13A5*
	rs9891398	17	*NF1*
	rs16950363	17	*CA10*
	rs9900961	17	*RPL17P41 | BPTF*
	rs6504840	17	*LOC100419014 | RPS2P48*
	rs8066872	17	*LINC00673*
	rs12947685	17	*COX11*
	rs12938347	17	*LINC01483 | LINC01028*
	rs2007530	17	*ARHGAP27P1*
	rs740642	17	***NTN1***
	rs7236390	18	*PIEZO2*
	rs630285	18	*AQP4-AS1*
	rs605961	18	*MPPE1*
	rs678570	18	***LAMA1***
	rs8091074	18	*LINC01387*
	rs12984574	19	*ZNF627*
	rs2288867	19	*ATP13A1*
	rs4807347	19	*ZNF555*
	rs17639568	19	*NFIX*
	**rs367209**	**19**	***LOC101928063***
	rs7252291	19	*CELF5 |* ***NFIC***
	rs760629	20	*PPIAP21 | EIF4EBP2P*

	rs13041524	20	***PLCB4***
	rs236114	20	*MCM8*
	rs6075924	20	*LOC284744 | LINC00261*
	rs6082789	20	*LNCNEF | KRT18P3*
	rs6014017	20	*PFDN4 | DOK5*
	rs8119892	20	*PPIAP21 | EIF4EBP2P*
	rs9679935	20	*VAPB*
	rs12152036	21	*MIR548XHG | PPIAP22*
	rs13048883	21	*C1QBPP | FDPSP6*
	rs4816257	21	*MRPL39*
	rs2257008	21	*MIR5009*
	rs7283527	21	*LOC101927869 | LINC01692*
	rs12484854	22	*LOC102724653*
	rs9612352	22	*ZDHHC8P1 |LINC01659*
	rs11703440	22	*LOC284898 | LINC02554*
	rs2298372	22	*DRICH1*
	rs5764804	22	***FBLN1***
	rs5760912	22	*CRYBB2*
	rs5752839	22	*ZNRF3*
	rs7288379	22	*SHISAL1 | LINC01656*
	rs9614616	22	*NUP50 | KIAA0930*
	rs11703546	22	*CPSF1P1 | RFPL3*
**APOEe4**	**rs367209**	**19**	***LOC101928063***
	**rs383133**	19	***ZNF221***
	**rs365745**	19	*ZNF221*
	**rs415499**	19	***ZNF155***

Table S3. ADNI-2 SNP signatures identified in the cases@control and in the APOEe4 tasks.
"# Chr" indicates the chromosome number and "|" in the Gene Symbol column indicates that specific SNP is located in an intergenic region between two genes.
In red color are highlighted those genes that are already known to be associated to AD. In bold black color are highlighted those genes/SNPs mentioned in the main manuscript.

GROUP	PATHWAYS
**1a**	Caspase activation via extrinsic apoptotic signaling pathway, intrinsic pathway for apoptosis, apoptosis execution phase, regulated necrosis, transmission across chemical synapse, amyloid fiber formation, deregulated CDK5 triggers multiple neurodegenerative pathways.
**1b**	Macroautophagy, cellular response to hypoxia, cellular response to heat stress, cellular senescence, detoxification of reactive oxygen species, potassium channels.
**1c**	Cellular senescence, detoxification of reactive oxygen species, PIP3 activates AKT signaling.
**2**	Metabolism of nitric oxide, mitochondrial protein import, mitochondrial iron-sulfur cluster biogenesis, the citric acid (TCA) cycle and respiratory electron transport, cellular senescence, detoxification of reactive oxygen species, mitochondrial translation, mitochondrial calcium ion transport.
**3**	Caspase activation via extrinsic apoptotic signaling pathway, intrinsic pathway for apoptosis, apoptosis execution phase, regulated necrosis, death receptor signaling
**4**	Clathrin-mediated endocytosis, translocation of GLUT4 to the plasma membrane, trans-golgi network vesicle budding, mitochondrial calcium ion transport, ABC-family proteins mediated transport, cellular hexose transport
**5a**	Amyloid fiber formation, unfolded protein response, regulation of insulin-like growth factor (IGF), mitochondrial protein import, chaperoning-mediated protein folding, post-chaperoning tubuling folding pathway, asparagin N-linked glycosilation, gamma carboxylation, carboxyterminal post-translation.
**5b**	Post-translation protein phosphorylation, neddylation, protein ubiquitination, deubiquitination, O-linked glycosilation, post-translational modification: synthesis of GPI-anchored proteins.
**6a**	Biological oxidation, the citric acid (TCA) cycle and respiratory electron transport, regulation of insulin secretion, glucagon signaling in metabolism regulation, metabolism of carbohydrates, digestion.
**6b**	Metabolism of nitric oxide, metabolism of lipids.
**7**	Cellular response to hypoxia, Cellular response to heat stress, detoxification of reactive oxygen species, cellular senescence, HSP90 chaperone cycle for steroid hormone receptors (SHR), cell junction organization, macroautophagy.
**8**	mTOR signaling, death receptor signaling, PIP3 activates AKT signaling, MAPK1/MAPK3 signaling, MAPK6/MAPK4 signaling, integrin signaling by leptin, integrin signaling by hippo, WNT ligand biogenesis and trafficking, degradation of beta-catenin by destruction complex, TCF dependent signaling in response to WNT, beta-catenin independent WNT signaling.
**9a**	GPCR ligand binding, GPCR downstream signaling, GASTRIN-CREB signaling, pre-NOTCH expression and processing, signaling by NOTCH1, signaling by NOTCH2, signaling by NOTCH3, signaling by NOTCH4.
**9b**	GPCR downstream signaling, GASTRIN-CREB signaling.
**9c2**	Signaling by TGF-beta family members.
**9c3**	Signaling by receptor tyrosine kinases.

Table S4. Groups of pathways selected in REACTOME and analyzed with Group Lasso with overlap in ADNI-1.

GROUP	PATHWAYS
**1a**	Caspase activation, intrinsic pathway for apoptosis, apoptosis execution phase, regulated necrosis, transmission across chemical synapse, amyloid fiber formation, deregulated CDK5 triggers multiple neurodegenerative pathways.
**1b1**	Cellular response to hypoxia, cellular response to heat stress, potassium channels.
**1b2**	Macroautophagy, detoxification of reactive oxygen species, cellular senescence.
**1c**	Cellular senescence, detoxification of reactive oxygen species, PIP3 activates AKT signaling.
**2**	Metabolism of nitric oxide, mitochondrial protein import, mitochondrial iron-sulfur cluster biogenesis, the citric acid (TCA) cycle and respiratory electron transport, cellular senescence, detoxification of reactive oxygen species, mitochondrial translation, mitochondrial calcium ion transport.
**3**	Caspase activation via extrinsic apoptotic signaling pathway, intrinsic pathway for apoptosis, apoptosis execution phase, regulated necrosis, death receptor signaling
**4**	Clathrin-mediated endocytosis, translocation of GLUT4 to the plasma membrane, trans-golgi network vesicle budding, mitochondrial calcium ion transport, ABC-family proteins mediated transport, cellular hexose transport
**5a**	Amyloid fiber formation, unfolded protein response, regulation of insulin-like growth factor (IGF), mitochondrial protein import, chaperoning-mediated protein folding, post-chaperoning tubuling folding pathway, asparagin N-linked glycosilation, gamma carboxylation, carboxyterminal post-translation.
**5b**	Post-translation protein phosphorylation, neddylation, protein ubiquitination, deubiquitination, O-linked glycosilation, post-translational modification: synthesis of GPI-anchored proteins.
**6a**	Biological oxidation, the citric acid (TCA) cycle and respiratory electron transport, regulation of insulin secretion, glucagon signaling in metabolism regulation, metabolism of carbohydrates, digestion.
**6b**	Metabolism of nitric oxide, metabolism of lipids.
**7**	Cellular response to hypoxia, Cellular response to heat stress, detoxification of reactive oxygen species, cellular senescence, HSP90 chaperone cycle for steroid hormone receptors (SHR), cell junction organization, macroautophagy.
**8**	mTOR signaling, death receptor signaling, PIP3 activates AKT signaling, MAPK1/MAPK3 signaling, MAPK6/MAPK4 signaling, integrin signaling by leptin, integrin signaling by hippo, WNT ligand biogenesis and trafficking, degradation of beta-catenin by destruction complex, TCF dependent signaling in response to WNT, beta-catenin independent WNT signaling.
**9a**	GPCR ligand binding, GPCR downstream signaling, GASTRIN-CREB signaling, pre-NOTCH expression and processing, signaling by NOTCH1, signaling by NOTCH2, signaling by NOTCH3, signaling by NOTCH4.
**9b**	GPCR downstream signaling, GASTRIN-CREB signaling.
**9c**	Signaling by TGF-beta family members, signaling by receptor tyrosine kinases.

Table S5 Groups of pathways selected in REACTOME and analyzed with Group Lasso with overlap in ADNI-2.

Supplemental Results

Validation of the SNP signatures

In order to verify the robustness of the identified SNPs signatures, a validation procedure was performed considering the dataset (ADNI-1 or ADNI-2) left available. Two steps characterize the validation: the first one consists in mapping the SNPs of the signature in another independent dataset, and the second one consists in analyzing the data matrix (having all the subjects and just the selection of SNPs of the identified list) evaluating the classification performance of the signature. When we started the validation procedure of the SNP signature identified in ADNI-1 cases@controls task, we tried to map all the 14 SNPs in ADNI-2 but just 9 SNPs were found. Despite this issue, we build the data matrix, having these 9 SNPs and 281 subjects, and we analyzed it using Regularized Least Square (RLS) classifier inside PALLADIO. Figure S2 shows that we could not validate this signature. A possible reason resides in the failure of the complete mapping of all the SNPs of our signature in ADNI-2.
In the validation procedure of the other ADNI-1 SNP signature, considering APOEe4 task, we encountered the same SNP mapping issue: in ADNI-2 we found just 24 SNPs over 39 total SNPs. Nonetheless this incomplete SNP mapping was sufficient to validate the signature in ADNI-2 (Figure S2).
When we tried to validate the SNP signatures identified in ADNI-2 in ADNI-1, we encountered the same SNP mapping issue explained before: for the cases@controls signature we found just 46 over 138 total SNPs and for the APOEe4 signature we found 2 over 4 total SNPs. Both these signatures did not pass the validation procedure (Figure S2).
Even if the validation procedure truly succeeded just for the APOEe4 signature identified in ADNI-1, we cannot state with certainty that the other three SNP signatures failed the validation because we were unable to map all the SNPs of these signatures in the validation dataset. A possible reason why we could validate just APOEe4 signature identified in ADNI-1, even if also in this case the SNP mapping was incomplete, could be found in the different "value" or "weight" of the SNPs of a signature. Since the SNPs are characterized by different weights, as confirmed the different risk of developing AD based on the number of copies of APOEe4, the probability that a SNP signature passes the validation will increase proportionally to the number and the weights of the mapped SNPs. Furthermore the method we chose to perform the SNP-based analysis (*i.e.,* l_1l_{2FS}) is designed to identify a list of features discriminant but also correlated. This last characteristic means that if the mapping procedure does not comprehends the most correlated SNPs, high is the probability that the validation of the signature will not succeed.

**Validation ADNI-1 in ADNI-2**	**cases@controls**			
	**B. ACC**	**p-value**	**MCC**	**p-value**
	0.51 ± 0.04	1.546e-01	0.03 ± 0.10	9.510e-02
	**APOEε4**			
	**B. ACC**	**p-value**	**MCC**	**p-value**
	0.74 ± 0.08	2.165e-23	0.65 ± 0.13	2.165e-23
**Validation ADNI-2 in ADNI-1**	**cases@controls**			
	**B. ACC**	**p-value**	**MCC**	**p-value**
	0.50 ± 0.05	6.779e-01	0.00 ± 0.10	5.077e-01
	**APOEε4**			
	**B. ACC**	**p-value**	**MCC**	**p-value**
	Na	*Na*	*Na*	*Na*

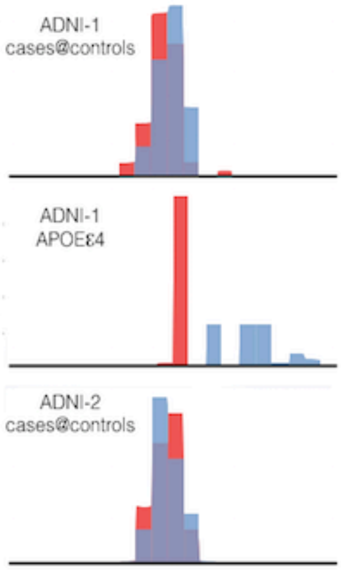

Figure S2. The validation results of the SNP signatures identifies in ADNI-1 and ADNI-2 dataset. In the validation procedure we consider the same two classification tasks: AD vs. healthy controls (cases@controls) and 1/2 APOEe4 vs. 0 APOEe4 carriers (APOEe4 task). B. ACC, Balanced Accuracy; MCC, Matthews Correlation Coefficient.

Supplementary References

1. Wu, M. C. *et al.* Rare-variant association testing for sequencing data with the sequence kernel association test. *Am. J. Hum. Genet.* **89**, 82–93 (2011).
2. Hastie, T., Tibshirani, R. & Wainwright, M. *Statistical learning with Sparsity : the lasso and generalizations*.
3. Barbieri, M., Fiorini, S., Tomasi, F. & Barla, A. PALLADIO: a parallel framework for robust variable selection in high-dimensional data. *Proc. 6th Work. Python High-Performance Sci. Comput.* 19–26 (2016). doi:10.1109/pyhpc.2016.13
4. Daniel, W. W. 'Kolmogorov-Smirnov one-sample test' in Applied Nonparametric statistics. in 635 (PWS-KENT Pub, 1990).
5. Wang, J., Duncan, D., Shi, Z. & Zhang, B. WEB-based GEne SeT AnaLysis Toolkit (WebGestalt): update 2013. *Nucleic Acids Res.* **41**, 77–83 (2013).
6. Kanehisa, M. & Goto, S. KEGG: Kyoto encyclopedia of genes and genomes. *Nucleic Acids Res.* **27**, 29–34 (1999).
7. Yu, W., Clyne, M., Khoury, M. J. & Gwinn, M. Phenopedia and genopedia: Disease-centered and gene-centered views of the evolving knowledge of human genetic associations. *Bioinformatics* **26**, 145–146 (2009).
